# Frontal–striatal connectivity and positive symptoms of schizophrenia: implications for the mechanistic basis of prefrontal rTMS

**DOI:** 10.1007/s00406-020-01163-6

**Published:** 2020-07-19

**Authors:** Roberto Limongi, Michael Mackinley, Kara Dempster, Ali R. Khan, Joseph S. Gati, Lena Palaniyappan

**Affiliations:** 1 Robarts Research Institute, 1151 Richmond St. N, UWO, London, ON N6A 5B7 Canada; 2 Department of Psychiatry, Schulich School of Medicine and Dentistry, University of Western Ontario, London, ON Canada; 3 Department of Psychiatry, Dalhousie University, Halifax, NS Canada; 4 Lawson Health Research Institute, London, ON Canada

**Keywords:** Dynamic causal modeling, Transcranial magnetic stimulation, Schizophrenia, Delusions

## Abstract

Repetitive transcranial magnetic stimulation (rTMS), when applied to left dorsolateral prefrontal cortex (LDLPFC), reduces negative symptoms of schizophrenia, but has no effect on positive symptoms. In a small number of cases, it appears to worsen the severity of positive symptoms. It has been hypothesized that high-frequency rTMS of the LDLPFC might increase the dopaminergic neurotransmission by driving the activity of the left striatum in the basal ganglia (LSTR)—increasing striatal dopaminergic activity. This hypothesis relies on the assumption that either the frontal–striatal connection or the intrinsic frontal and/or striatal connections covary with the severity of positive symptoms. The current work aimed to evaluate this assumption by studying the association between positive and negative symptoms severity and the effective connectivity within the frontal and striatal network using dynamic causal modeling of resting state fMRI in a sample of 19 first episode psychosis subjects. We found that the total score of positive symptoms of schizophrenia is strongly associated with the frontostriatal circuitry. Stronger intrinsic inhibitory tone of LDLPFC and LSTR, as well as decreased bidirectional excitatory influence between the LDLPFC and the LSTR is related to the severity of positive symptoms, especially delusions. We interpret that an increase in striatal dopaminergic tone that underlies positive symptoms is likely associated with increased prefrontal inhibitory tone, strengthening the frontostriatal ‘brake’. Furthermore, based on our model, we propose that lessening of positive symptoms could be achieved by means of continuous theta-burst or low-frequency (1 Hz) rTMS of the prefrontal area.

## Introduction

Neuromodulatory interventions such as repetitive transcranial magnetic stimulation (rTMS) are appropriate when antipsychotic treatment fails to control psychotic symptoms [40]. While rTMS applied to left dorsolateral prefrontal cortex (LDLPFC) reduces the burden of negative symptoms [22, 39] in schizophrenia, a recent review has highlighted that, in some cases, high-frequency left prefrontal rTMS could worsen the severity of positive symptoms [20]. In this work, we explore the mechanistic basis of this phenomenon, and expound a circuit-based model to treat positive symptoms, which are currently not clinical targets for rTMS therapy.

When treating psychosis, the effect of rTMS strongly varies with the site of stimulation. Auditory hallucinations decrease upon high-frequency stimulation (HF-rTMS) of the left temporo-parietal junction [30], while left prefrontal HF-rTMS appears effective to treat negative symptoms. In contrast, while HF-rTMS of TPJ has no effect on negative symptoms, stimulating the LDLPFC appears to worsen positive symptoms in some patients [20]. Though individual studies reporting worsening of positive symptoms [12] have not identified if this effect is specific to certain positive symptoms, the earliest anecdotes indicated a specific but brief detrimental effect on delusions [44, 56]. More recent trials indicate that some positive symptoms such as excitement may indeed improve with HF-rTMS of LDLPFC [13]. Importantly, out of 11 controlled trials investigating the effect of prefrontal HF-rTMS, worsening of positive symptoms have been reported in only some (e.g., [22]), as highlighted by Kennedy et al. [20] and Marzouk et al. [27]. Similarly, the extensive literature on HF-rTMS to LDLPFC in depression does not indicate any increase in the risk of positive psychotic symptoms [36]. Taken together, these observations indicate that a subset of patients with schizophrenia is likely to have a brief exacerbation of certain positive symptoms when HF-rTMS is applied to LDLPFC.

Seminal combined TMS/positron emission tomography (PET) studies have indicated the possibility of striatal dopamine release in response to prefrontal stimulation in humans [21, 46]. Carlsson [3] outlined the possibility of a pyramidal glutamatergic frontostriatal feedback accelerator circuit that facilitates striatal dopaminergic output, which, when excessive, is balanced by a GABAergic inhibitory brake circuit. Based on these studies, and in keeping with the longstanding dopaminergic hypothesis of positive symptoms of schizophrenia [14], Kennedy et al. [20] hypothesized that the apparent worsening of positive symptoms on LDLPFC HF-rTMS is likely due to disrupted prefrontal excitation–inhibition balance [28], leading to left dorsal striatal dopaminergic excess via the frontostriatal network.

In this study, we further parse the hypothesis proposed by Kennedy et al. [20] and test if the intrinsic frontal connections (reflecting the inhibition/excitation balance) and the frontal–striatal connectivity (reflecting interregional influences) relate to the severity of positive symptoms. We pursued this evaluation by using dynamic causal modeling (DCM) [6] of resting state fMRI in a minimally medicated, actively symptomatic sample of patients with first episode psychosis (FEP). As briefly summarized below, DCM emerges as a physiologically realistic tool for evaluating this assumption, thus explaining the mechanistic basis of symptom exacerbation after prefrontal HF-rTMS and on the other hand providing insight into the parameters of LDLPFC stimulation that can be therapeutically beneficial for the same symptoms.

## Dynamic causal modeling of network connectivity

In dynamic causal modeling, differential equation models of “neural states” are fit to time series data. In the case of fMRI, the main goal of DCM is to make inferences about the neural causes of the BOLD signals in a priori-defined brain regions observed during either resting state [35] or task execution [6]. This allows the researcher or clinician to quantitatively infer how the time series is generated by (unobserved) neural activity of coupled neuronal populations.

At present, DCM is considered the most physiologically grounded technique to infer the effective connectivity between brain regions [9]. The particular neural model we use here treats each brain region as having coupled excitatory and inhibitory neural populations, with long distance excitatory connections between regions. This arrangement, typically found in neural mass models, is based on the finding that within-region (intrinsic) connections primarily relate to inhibitory (GABAergic) interneurons, whereas extrinsic connections are typically glutamatergic. Importantly, model parameters are estimated via Bayesian inference [7]. Therefore, researchers can fit different models (each model representing one hypothesis about how the fMRI data were generated) [54, 55] and, crucially, investigate how the effective connectivity covaries with, for example, symptoms severity and clinical interventions (e.g., rTMS).

In this work, we capitalized on the utility of DCM to shed light on why positive symptoms of schizophrenia could exacerbate upon applying HF-rTMS to the LDLPFC. We fit 12 models corresponding to core symptoms of PANSS-8 scale to resting-state fMRI data obtained at ultra-high (7T) field from 19 FEP subjects. Using Bayesian model selection, we determined which model (i.e., symptoms metric) better explains the between-subjects variability in connections. Given the anecdotal evidence as discussed above, we expected the severity of positive symptoms, rather than negative symptoms, to relate to the frontostriatal effective connectivity [47]. In addition, given that only a subset of patients is likely to experience exacerbated positive symptoms, we aim to identify the connectivity pattern that can predict this adverse outcome of prefrontal HF-rTMS in schizophrenia.

## Methods and materials

### Subjects

Nineteen FEP subjects participated in the study (Table 1). This patient sample has been previously reported in [25]. Subjects were recruited from the “Prevention and Early Intervention Program for Psychosis” in London, Ontario. Criteria for inclusion in the FEP group included (1) first clinical presentation with psychotic symptoms and DSM-5 [2] criteria A for schizophrenia satisfied; (2) less than 2 weeks of lifetime antipsychotic exposure. Roughly 40% of patients were not exposed to any antipsychotic at the time of assessment (Table 2). Defined Daily Dose exposure was calculated for these patients and the mean antipsychotic Defined Daily Dose in our sample was 1.05 (suggesting the average patient had had 1-day worth of the minimum maintenance dose at the time of assessment).

**Table 1:** Demographic information of participants

Subject	Ethnicity	SOFAS	Cannabis use (self-endorsed)	Parental SES (NSSEC)	Age at study date	Gender	Age psychosis onset (years)	Duration of untreated psychosis (months)
1	C	40	1	4	19	Male	17	24
2	C	37	1	5	20	Male	20	4
3	C	40	1	2	19	Male	19	1
4	C	60	1	2	17	Male	17	2
5	C	30	1	4	18	Male	18	2
6	C	51	0	4	17	Female	16	9
7	A/ME	34	0	5	24	Male	23	12
8	C	50	1	2	21	Male	21	1
9	C	25	1	2	25	Male	20	59
10	A/ME	40	1	2	28	Male	28	3
11	C	33	1	2	20	Female	18	14
12	C	65	0	3	23	Female	N/A	N/A
13	A/ME	25	1	2	23	Male	22	6
14	C	44	1	3	24	Female	24	1
15	C	20	0	2	23	Female	N/A	N/A
16	C	55	1	4	20	Male	19	9
17	C	50	1	1	27	Male	21	72
18	C	40	0	5	26	Female	26	1
19	C	45	1	4	19	Female	19	1

Age FEP male *M* = 21.75, SD = 3.59; age FEP female *M* = 21.71, SD = 3.15

*C* Caucasian, *A/ME* Asian or Middle Eastern, *NSSEC* National Statistics Socioeconomic Status, *SES* Socioeconomic Status, *SOFAS* Social and Occupational Functioning Assessment Scale, *N/A* non-reliable information

**Table 2:** Participants’ medication at the day of assessment

Subject	Days of antipsychotic	Antipsychotic	Dose (mg)	Duration (days)	Defined daily dose (DDD)
1	0	0	0	0	0
2	0	0	0	0	0
3	7	Paliperidone	6	7	7
4	0	0	0	0	0
5	7	Aripiprazole	5	7	2.6
6	0	0	0	0	0
7	0	0	0	0	0
8	0	0	0	0	0
9	7	Paliperidone	3, 6	3, 4	5.5
10	0	Olanzapine	10	1	1
11	0	0	0	0	0
12	5	Risperidone	1.5	5	1.5
13	0	0	0	0	0
14	7	Olanzapine	7.5	7	5.25
15	0	0	0	0	0
16	0	0	0	0	0
17	0	0	0	0	0
18	7	Risperidone	1	7	1.4
19	10	Aripiprazole	5	10	3.333

All patients with FEP received a consensus diagnosis from 3 psychiatrists (LP/KD and the primary treatment provider) after approximately 6 months on the basis of the best estimate procedure, as described in [24], and the Structured Clinical Interview for DSM-5. All patients satisfied criteria for Schizophrenia-Spectrum Disorders, with 15 patients satisfying DSM-5 criteria for Schizophrenia and 3 for Schizoaffective Disorder. One subject lacked follow-up clinical data at 6 months, with the available baseline data suggesting a diagnosis of Schizophreniform Disorder. We use the term FEP to describe the patient group to capture all the schizophrenia-spectrum disorders as above. Informed consent from participants was obtained according to the approval by Western University’s Human Ethics Committee. Symptoms assessment was performed using Positive and Negative Syndrome Scale-8 items version ([1], Table 3). In this sample, subjects predominantly showed positive symptoms, mean difference between total positive and total negative symptoms = 5.21, SD = 4.44, 95% CI [3.07, 7.35], *t*(18) = 5.11, *p* < 0.0001.

**Table 3:** Symptoms scores assessed via the 8-item positive and negative symptoms scale (PANSS-8)

Subject	PANSS-8								PANSS negative	PANSS positive	PANSS-8 total
	P1	P2	P3	N1	N4	N6	G5	G9			
1	4	4	4	4	5	3	2	4	12	12	30
2	5	4	5	2	3	1	3	4	6	14	27
3	4	5	4	5	4	3	1	3	12	13	29
4	5	1	5	3	4	3	1	4	10	11	26
5	5	3	4	2	3	3	1	3	8	12	24
6	5	1	5	3	5	3	3	2	11	11	27
7	6	5	6	1	1	1	4	5	3	17	29
8	5	4	4	2	4	2	1	4	8	13	26
9	7	3	5	4	3	1	2	6	8	15	31
10	6	4	2	1	1	1	1	3	3	12	19
11	6	3	2	1	1	1	1	4	3	11	19
12	4	2	5	3	1	2	1	3	6	11	21
13	5	6	5	1	3	4	3	5	8	16	32
14	5	4	2	1	1	1	1	5	3	11	20
15	7	4	6	1	1	1	1	6	3	17	27
16	5	1	5	4	3	1	1	3	8	11	23
17	7	3	2	5	4	3	1	3	12	12	28
18	5	1	5	1	5	1	1	3	7	11	22
19	5	3	1	3	3	3	1	4	9	9	23

*P1* delusions, *P2* conceptual disorganization, *P3* hallucinations, *N1* blunted affect, *N4* social withdrawal, *N6* lack of spontaneity, *G5* mannerisms, *G9* unusual thoughts)

### Resting-state fMRI

All data were acquired using a 680-mm neuro-optimized 7-T MRI (Siemens MAGNETOM Plus, Erlangen, Germany) equipped with an AC84 II head gradient coil and an 8-channel Tx, 32-channel Rx radiofrequency coil. We acquired 360 resting-state whole-brain functional images over 6 min. A gradient echo-planar-imaging sequence was used with an echo time (TE) = 20 ms, repetition time (TR) = 1000 ms, flip angle = 30 deg, field of view = 208 mm, voxel dimension = 2 mm isotropic and 63 contiguous slices. The EPI data were accelerated using GRAPPA = 3 and a multi-band factor = 3. A 3D T1-weighted MP2RAGE anatomical volume (TE/TR = 2.83/6000 ms, TI1/TI2 = 800/2700 ms) at 750 µm isotropic resolution were acquired as an anatomical reference.

### Effective connectivity

#### Spectral DCM

At a subject level, we estimated the resting-state effective connectivity within the LDLPFC–LSTR network by fitting a fully connected two-state spectral dynamic causal model [26] to the fMRI data [35]. Functional images were realigned, normalized to the Montreal Neurological Institute (MNI) space, and spatially smoothed using a 4 mm (full width at half maximum) Gaussian kernel. We fit a general linear model to the images and included six head movement parameters and time series corresponding to the white matter and cerebrospinal fluid as regressors. We included a cosine basis set with frequencies ranging from 0.0078 to 0.1 Hz [17]. Images were high-pass filtered to remove slow-frequency drifts (< 0.0078 Hz).

**Fig. 1:**
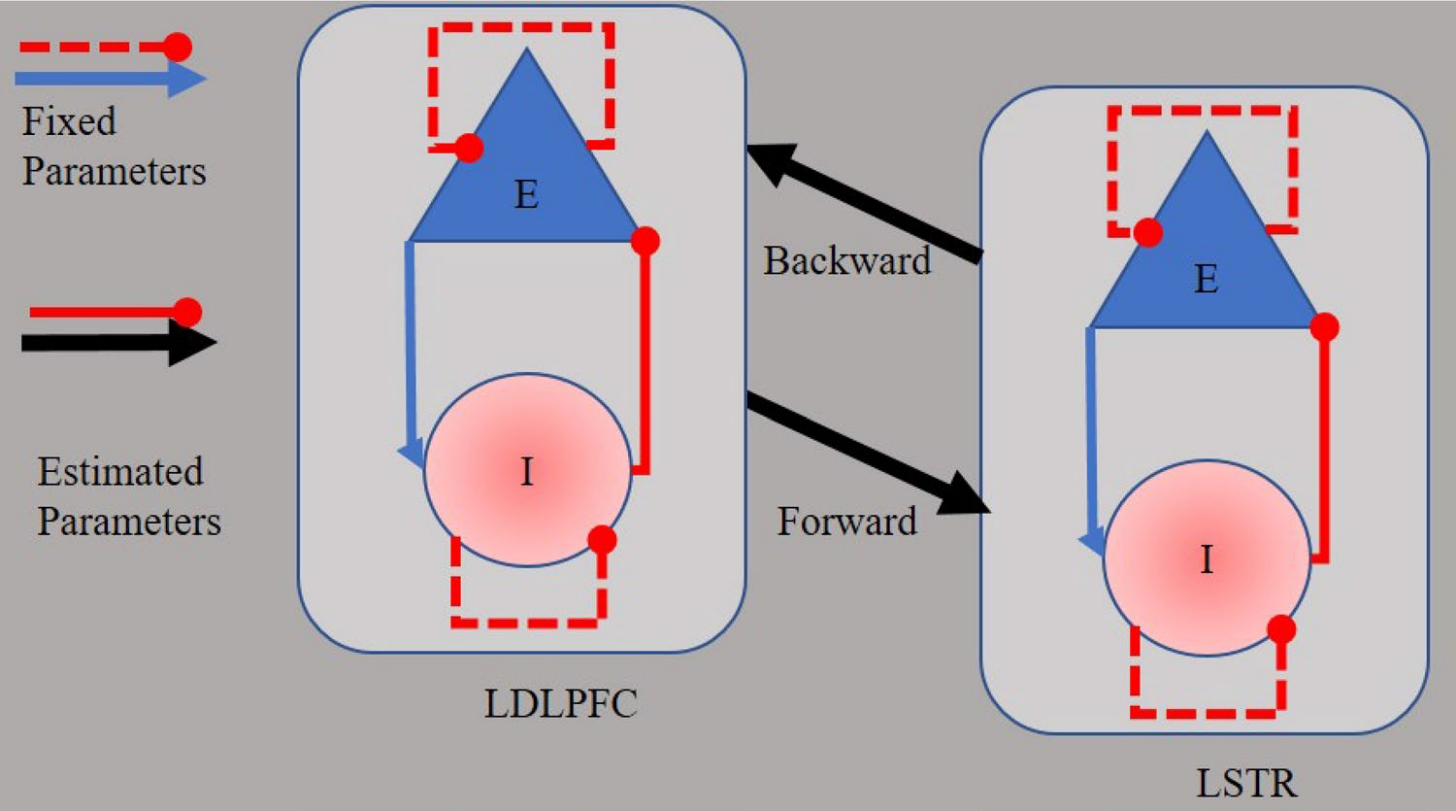
Two-state dynamic causal model of the frontostriatal network. Each region comprises one population of excitatory neurons (E) and one population of inhibitory neurons (I). Parameters of effective connectivity represent the influence of inhibitory to excitatory connections (IE, assumed to be GABAergic neurons), the influence of excitatory to inhibitory connections (EI), the influence of self-inhibitory connections within each population (SE, SI), and the influence of excitatory population of one region on the excitatory population of the other region (EE, assumed to be glutamatergic connections). Whereas EI, SE, and SI parameters are fixed in the model, IE and EE are free to vary, evaluated parameters

We identified regions with blood oxygen level fluctuations within frequencies ranging from 0.0078 to 0.1 Hz [17] via an F-contrast. We extracted the time series that summarized the activity within spheres (8-mm radius) in the LDPFC (MNI coordinates *x* = − 35.5 *y* = 49.4 *z* = 32.4 [31] and in the LSTR (MNI coordinates *x* = − 13, *y* = 15, and *z* = 9), [37, 52]. Two-state DCM assumes excitatory and inhibitory populations of neurons within a region. Each population comprises self-inhibition connections. Crucially, two free parameters are fit to the fMRI data: interregional excitatory-to-excitatory connections and within-region inhibitory-to-excitatory connections (Fig. 1). Each of these parameters is the log of a scaling factor, which is multiplied by the default connection strength: 1/8 Hz for between-region connections and − 1/8 Hz for within-region connections. This formulation enforces positivity or negativity constraints on the connections, and gives the parameters a simple interpretation, as follows. Between-region connections are excitatory, so more positive values correspond to greater excitation and more negative values correspond to less excitation. Within-region connections are inhibitory, so more positive values indicate greater inhibition and less positive values indicate less inhibition.

#### Parametric empirical Bayes (PEB)

At a group level, we relied on PEB [5, 8, 55] to estimate connectivity parameters and the effect of covariates (e.g., symptoms) on these parameters. This complements the dynamic model of neural coupling at the within-subject level with a linear regression model of the connectivity parameters at the between-subjects level—which is estimated using Bayesian methods. Defining each candidate PEB model therefore requires selecting the between-subject regressors or covariates of interest.

We estimated 9 models aiming to assess the evidence in support of the hypothesis that positive symptoms measured using the PANSS-8 scale best explained the effective connectivity within the two-node network. We searched for evidence in support of positive symptoms relative to evidence either in support of negative symptoms or in support of the no association between symptoms and the network’s effective connectivity. Therefore, the model space comprised nine models which were labeled as follows: delusions (P1), disorganization (P2), hallucinations (P3), total positive (P1 + P2 + P3), blunted affect (N1), social withdrawal (N4), lack of spontaneity (N6), total negative (N1 + N4 + N6), and null. The design matrix of each model comprised a constant (column of ones) followed by the covariate of interest (symptoms). Furthermore, all but the null model included two covariates of no interest. The first covariate was a (binary; yes, no) regressor indicating whether a given subject received any medication. As suggested by one of the reviewers, who raised the issue of the possible confound of DUP on the relationship between symptoms and connectivity, we included DUP as the second covariate of no interest in our analysis. The design matrix of the null model comprised only a column of ones.

#### Group-level models’ evidence (reliability)

In PEB, subjects’ posterior estimates that were estimated using uninformative priors are used as empirical priors to estimate posteriors at the group level. Crucially, these second-level posteriors are sent back to the (subjects’ first level) as informative priors upon which subject’s posteriors are re-estimated. In other words, the estimation of group-level parameters (i.e., posterior estimates) rests on the optimization of priors at a subject level. This has one important consequence. It allows using small sample size in psychiatry studies—where getting a large sample of patients is challenging.^1^ This is simply because the less data (i.e., subjects) we have, the more affected the posteriors are by the priors. Therefore, with a small sample size the PEB scheme provides more informed priors, ensuing more precise posteriors and crucially maximizing the model evidence. As we detail below, this recursive scheme of PEB also has an important consequence in model selection.

#### Model comparison and selection

To test the hypothesis that positive symptoms affect the effective connectivity of our two-node network, we relied on Bayesian model comparison [33]. With this procedure, we assessed the (PEB-maximized) evidence of every model (approximated by the negative variational free energy, *F*). Basically, the model with the least negative free energy is the model with the strongest evidence. However, in practice it is useful to assess the evidence of a given model relative to the evidence afforded to the competing models. Bayesian model comparison achieves this by comparing the evidence of a given model (F1) with the evidence of the model with the most negative free energy (F2), yielding the log of the Bayes factor (ln BF_1_ = *F*_1_ − *F*). BF > 20 (i.e., ln BF ≈ 3) is equivalent to a posterior probability PP > 0.95 [33] which grants the model very strong evidence and is used as threshold for model selection [18]. Furthermore, by application of Bayes rule, the log Bayes factor can be converted to a posterior probability for each model—which sums to 1 across all models. Note that although the model evidence of a specific model is independent of the model space (since the evidence of each competing model is indexed by the negative variational free energy), the probabilities of all models in the comparison must sum to one. Therefore, the posterior beliefs about which model produced the fMRI data depends on the model space [45].

## Results

At a subject level, Table 4 shows the model diagnostics in terms of the percentage of explained variance, *M* = 25.01 (SD = 14.22), a value of at least 10% explained variance (averaged across subjects) is considered acceptable [54]. This was the case for all but two subjects. However, we could be confident that those two subjects did not make a large contribution to the group-level results because PEB weights the contribution of each subject by the posterior precision (confidence) of their parameters. Table 5 shows the parameter estimates of each subject’s model.

**Table 4:** Model diagnostics

Subject	% of variance accounted for by the model
1	38.08
2	13.44
3	44.32
4	25.54
5	13.14
6	7.22
7	10.25
8	1.78
9	35.28
10	50.95
11	25.25
12	43.86
13	20.08
14	37.83
15	30.3
16	27.59
17	11.42
18	11.02
19	27.87

**Table 5:** Parameter estimates of connectivity strength at a subject level

Subject	Connection			
	LDLPFC → LDLPFC	LSTR → LDLPFC	LDLPFC → LSTR	LSTR → LSTR
1	6.663	0.856	0.848	7.744
2	2.527	0.846	0.769	7.297
3	6.910	0.857	0.852	7.500
4	3.664	0.831	0.762	7.715
5	2.483	0.850	0.771	8.438
6	1.899	0.869	0.780	8.414
7	2.172	0.856	0.764	8.703
8	1.283	0.908	0.815	8.240
9	6.897	0.855	0.858	6.773
10	7.499	0.856	0.855	7.594
11	3.819	0.831	0.772	7.336
12	7.461	0.849	0.854	6.845
13	3.102	0.844	0.766	7.576
14	7.066	0.855	0.858	6.916
15	7.518	0.839	0.852	6.215
16	5.636	0.853	0.832	7.724
17	2.681	0.818	0.757	6.391
18	2.217	0.820	0.718	8.018
19	5.615	0.847	0.822	7.787

The estimates are the exponential of the connectivity matrix, exp (DCM.Ep.A), which scales the priors. Values greater than 1 represent a larger effect than the prior (i.e., an increase in the connectivity strength), whereas values smaller than 1 represent a smaller effect than the prior (i.e., a decrease in the connectivity strength)

**Fig. 2:**
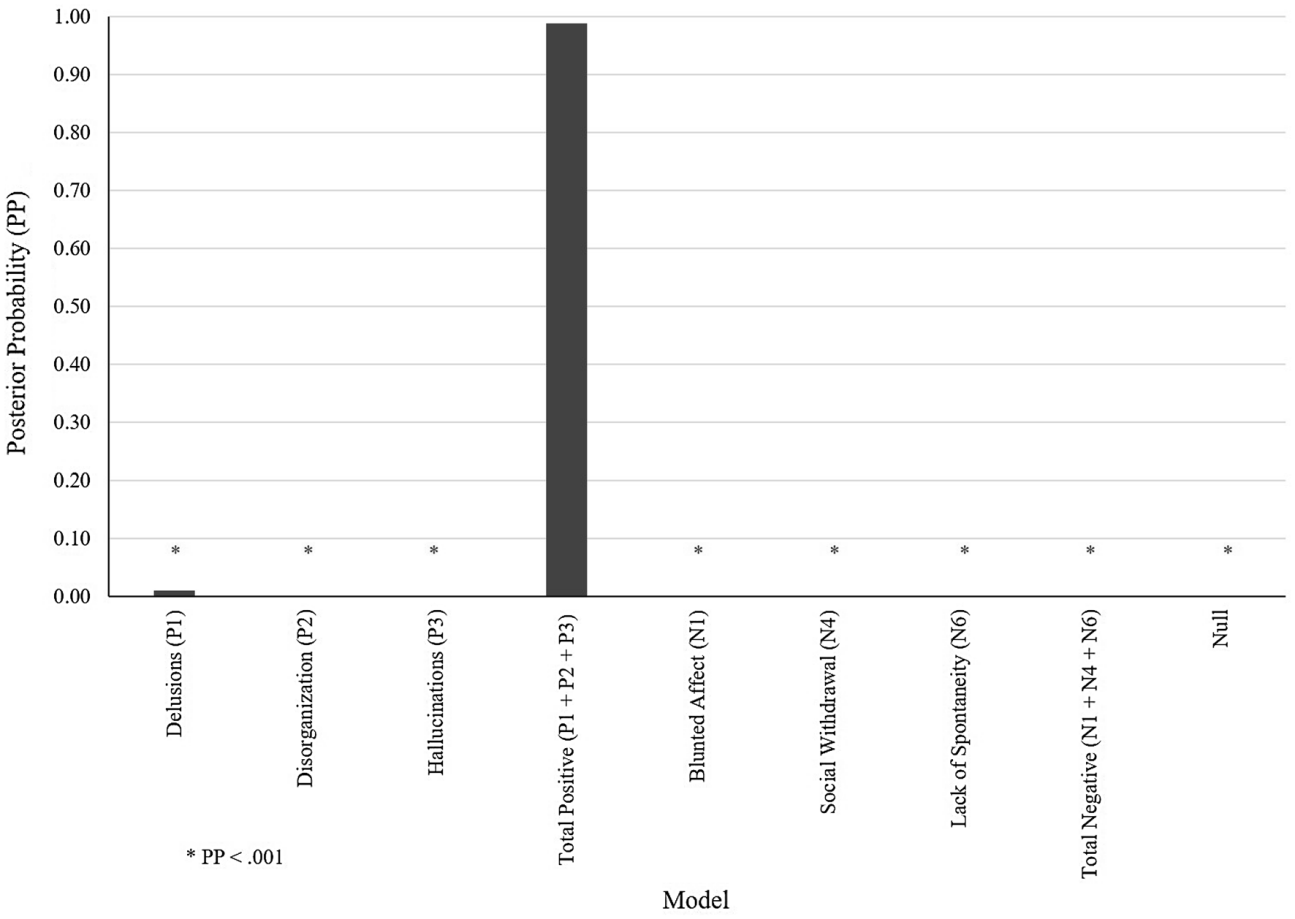
Model space and Bayesian model comparison results. Each model represented the association between one type of symptoms and the effective connectivity within the fronto striatal network. The null model represented no association between symptoms and connections

**Fig. 3:**
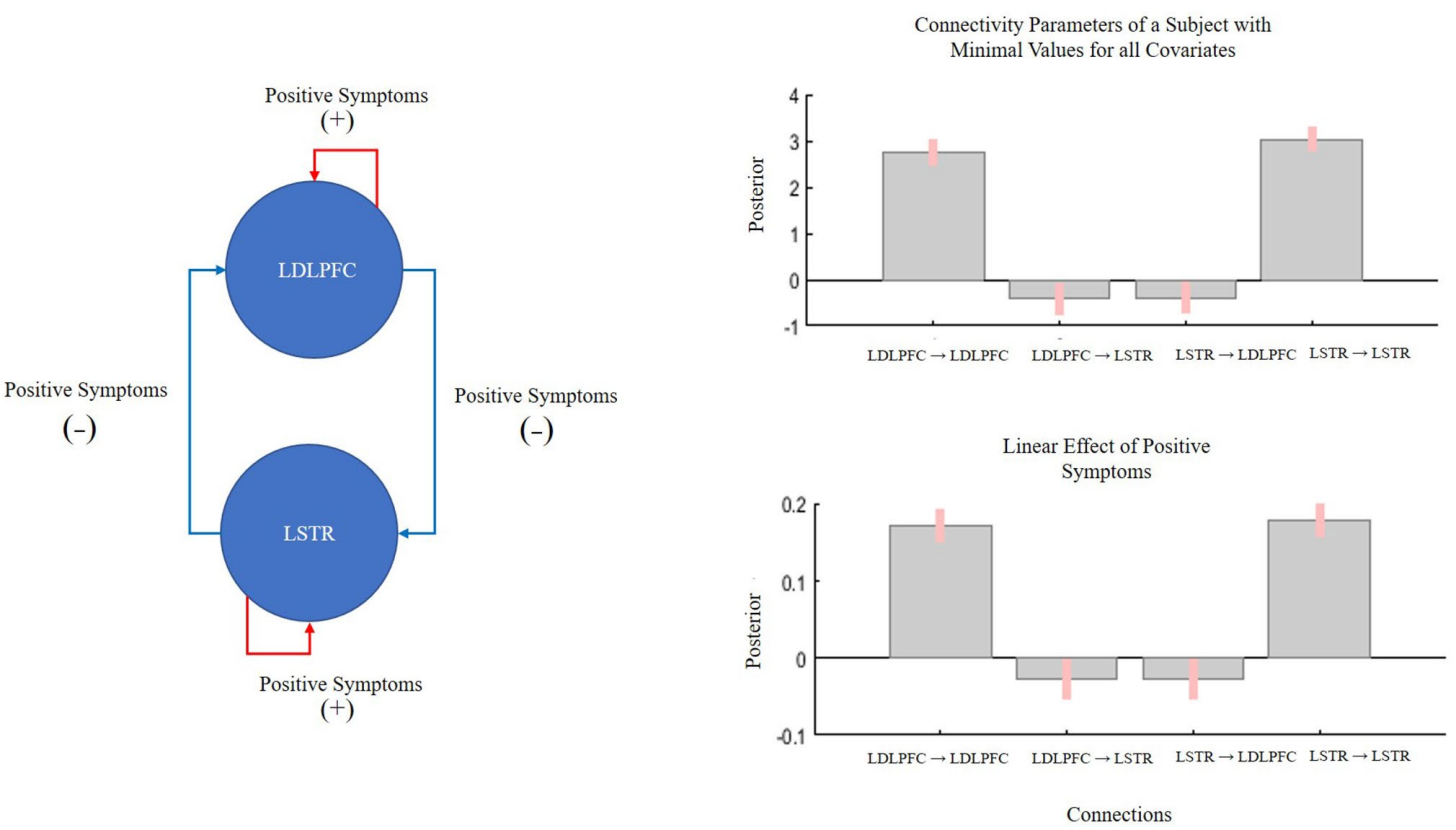
Parameter estimates of the winning (delusions) model. Bar graphs represent the rate of change of connectivity strength of a subject with the lowest values of all covariates (top) and the linear effect of positive symptoms—after accounting for all covariate of interests—across subjects. Left circuit shows a visual representation of the effect of positive symptoms on connectivity strength. Symptoms severity increases within region GABAergic activity and decreases between-region glutamatergic influence

At a group level, Bayesian model comparison revealed that the model representing the effect of total positive scores on the network’s effective connectivity outperformed all other models, posterior probability (PP) > 0.95 (Fig. 2), followed by delusions. Table 6 shows the resulting free energy of each model after maximizing its evidence. Crucially, in the winning model positive symptom severity affected all connections of the circuit (Fig. 3). Both LDPFC → LSTR and LSTR → LDPFC connectivity strength decreased with the severity of positive symptoms (PP = 0.96, and PP = 0.95, respectively). Furthermore, inhibitory activity within both the LDLPFC and the LSTR increased with positive symptom severity (PP = 1).

**Table 6:** Models’ evidence (negative variational free energy)

Symptom model	Free energy
Delusions (P1)	− 5966
Disorganization (P2)	− − 6012
Hallucinations (P3)	− 6016
Total positive (P1 + P2 + P3)	− 5961
Blunted affect (N1)	− 6045
Social withdrawal (N4)	− 6068
Lack of spontaneity (N6)	− 6043
Total negative (N1 + N4 + N6)	− 6040
Null	− 6155

The PEB scheme granted the least negative free energy to the “Total positive (P1 + P2 + P3)” model, followed by the Delusions (P1)

## Discussion

In this work, we aimed to elucidate the mechanism of exacerbation of positive symptoms of schizophrenia upon applying HF-rTMS to the LDLPFC. To this aim, we evaluated the hypothesis that the frontal–striatal connectivity underlies the severity of positive symptoms of schizophrenia. This would allow us to provide an empirical framework to design rTMS paradigms that can effectively reduce positive symptoms. We have two major findings. (1) In the absence of task-related demands (i.e., at ‘rest’), in patients in early stages of schizophrenia, the frontostriatal connectivity characterized by intrinsic inhibitory connections as well as exogenous forward (from the LDLPFC to the LSTR) and backward (from the LSTR to the LDLPFC) connections, relate to the burden of positive symptoms, irrespective of the influence of medication and DUP). (2) Patients with more severe burden of positive symptoms had a strong inhibitory tone within the LDLPFC and LSTR and reduced bidirectional exogenous excitatory tone. As we elaborate upon below, these observations might (1) speak to the likely nature of glutamate–dopamine interactions in frontostriatal circuit in schizophrenia and (2) provide a framework for future TMS studies to address positive symptoms in treatment-resistant patients.

Even in the subject with the lowest values of both positive symptoms and covariates of no interest (the intercept of the regression model, Fig. 3), we observed a bidirectional, predominantly decreased influence in the exogenous connections between LDLPFC and LSTR in our sample. This indicates that at resting state, the indirect GABA-mediated frontostriatal ‘brake’ pathway is likely to be dominant [3, 38], with any increase in prefrontal excitation poised to reduce striatal activity. This observation is congruent with combined PET/MRS studies indicating an inverse relationship between prefrontal glutamate and striatal dopamine levels in healthy controls [10] as well as patients with schizophrenia [16]. Our data do not provide direct information on the striatal dopaminergic activity in resting state. However, the observed decreased influence from the LDLPFC to the LSTR (i.e., frontostriatal influence) allows us to speculate that when dopaminergic excess occurs in the striatum, enhancing prefrontal ‘brakes’ on the striatum may have a desirable effect of reducing striatal hyperdopaminergia. As a corollary, any reduction in frontostriatal inhibition may have the effect of switching from ‘brake’ to ‘accelerator’ mode, further enhancing the hyperdopaminergic state [23].

In patients with severe positive symptoms, we noted an increase in prefrontal and striatal inhibitory tone, and decreased frontostriatal influence (Fig. 3). This pattern is consistent with enhanced, GABA-mediated, indirect frontostriatal pathways (i.e., stronger brakes). Given that this sample is (mostly) of unmedicated (or scarcely medicated) first episode subjects who responded well to antipsychotics in the next 6 months (16 out of 19 achieved > 50% reduction in overall PANSS-8 scores when clinically followed-up), we favor the speculation that the observed connectivity patterns are secondary to higher striatal dopaminergic activity. Thus, enhanced self-inhibitory tones of LDLPFC and LSTR, and a strong frontostriatal ‘brake’ are likely to be compensatory changes, albeit inefficient.

Based on the above, reducing LDLPFC’s inhibitory tone (i.e., disinhibition via NMDA blockade), may release the brakes and likely worsen positive symptoms. This framework provides a mechanistic explanation as to why those rTMS protocols that cause disinhibition (e.g., > 5–10 Hz) may worsen positive symptoms. While this conjecture is based on the glutamate hypothesis [4, 25, 32, 41, 42], our data neither supports nor refutes the primacy of glutamate over dopaminergic dysfunction in psychosis.

It is important to note that we purposefully studied an acutely ill, untreated first episode sample; thus P1 ratings were between 4 and 7. Increasing the sample size is unlikely to have an impact on the range of values in P1, as by default, untreated acute psychosis is characterized by such a high degree and narrow severity range of positive symptoms. With total positive symptoms (P1 + P2 + P3), the range was larger, with summed scores varying between 9 and 17. Both total positive score model and P1 had the best model evidence in our PEB analysis.

In the RESIS trial [53], the primary endpoint of negative symptoms did not improve with HF-rTMS of LDLPFC. Interestingly, an exploratory re-analysis showed improvement in non-specific factors including excitement and general psychopathology [13]. Furthermore, a subsequent sub-group analysis in clozapine-exposed patients revealed a surprising beneficial effect on positive symptoms, as well as general and total psychopathology but not negative symptoms [51]. In fact, individual patient-data meta-analysis in clozapine-exposed TRS patients failed to replicate the beneficial effect reported in RESIS trial [50]. Unlike subjects in Wagner et al.’s report [51], our subjects were not treatment resistant and were not exposed to clozapine. Treatment resistance status, especially clozapine resistance, is linked to a specific lack of striatal hyperdopaminergic state [34]. In such a normodopaminergic state, ‘releasing frontostriatal brakes’ by HF-rTMS of LDLPFC may not worsen positive symptoms; in fact, this may be beneficial in reducing excitement and total psychopathology, as observed in the RESIS study [13]. Furthermore, clozapine is suspected to have dissociable effects on GABA receptor subsystems, which may interact with the physiological response to rTMS [19].

### Inhibitory rTMS in the left DLPFC could lessen positive symptoms

**Fig. 4:**
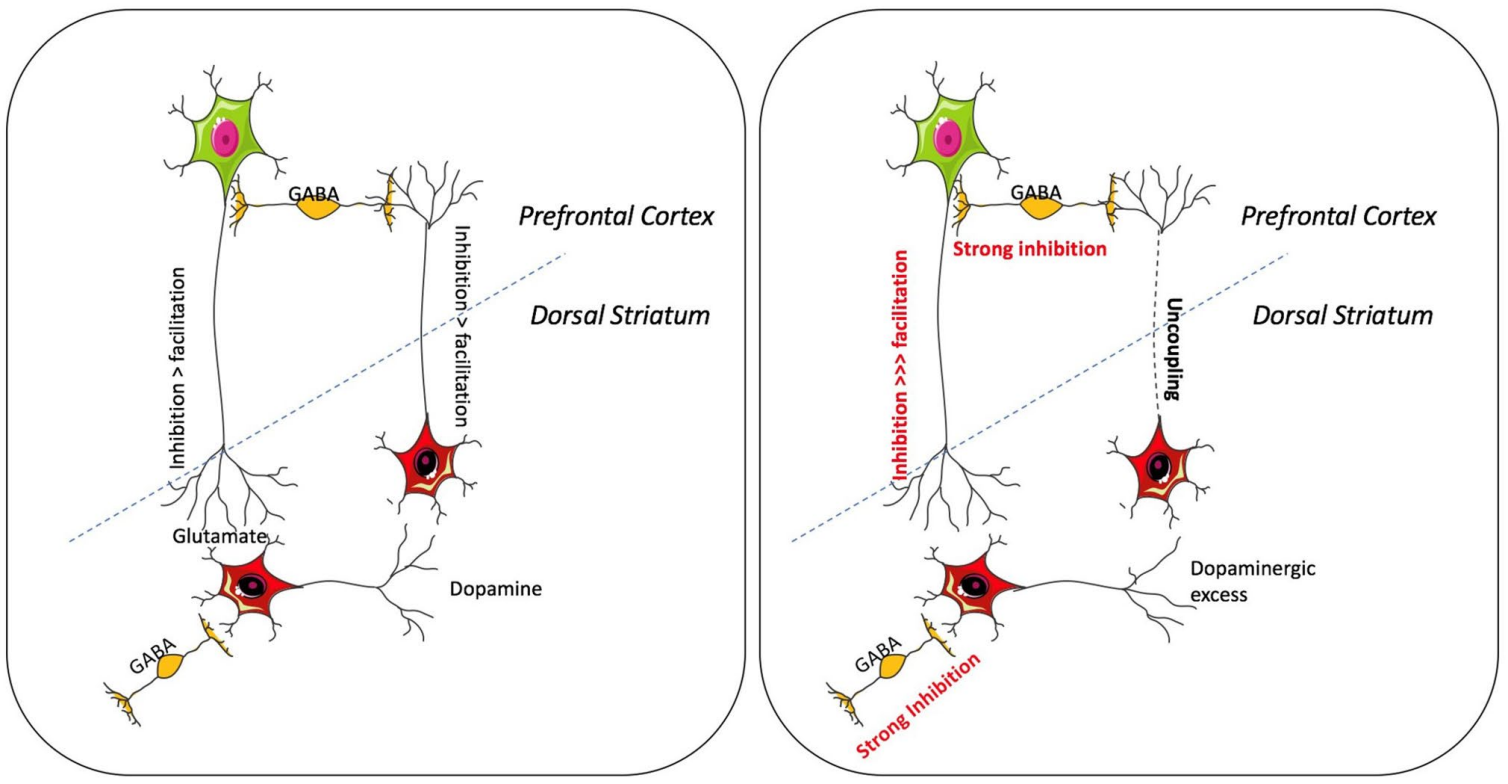
Frontostriatal connectivity and positive symptoms. The left panel represents the bidirectional inhibitory exogenous connections presumably of glutamatergic (from dorsolateral prefrontal cortex) and dopaminergic (from striatum) nature, and GABAergic inhibitory neuronal populations within both structures. The right panel represents the state of the circuit in response to striatal hyperdopaminergic tone, with stronger inhibitory tone in both striatal and prefrontal inhibitory neuronal populations. Furthermore, the frontostriatal brakes are now stronger, and this may be essential to achieve a satisfactory response to treatment of positive symptoms. The dashed blue lines indicate the cortex/sub-cortex boundary, distinguishing LDLPFC and the LSTR. Illustrations developed from the images provided by https://smart.servier.com/

The DCM results lead us to hypothesize that protocols that increase the inhibitory tone on cortical pyramidal neurons, may reduce further striatal dopaminergic output and thus reduce positive symptoms (Fig. 4). We have previously demonstrated that intermittent theta-burst stimulation (iTBS) reduced GABA/glutamate ratio in LDLPFC in healthy controls, tilting the balance towards reduced self-inhibitory tone [15]. Thus, LDLPFC iTBS can potentially increase overall excitatory output from the stimulated cortex thus abolishing the “brakes”. In contrast, continuous TBS has been shown to increase GABA (albeit in the motor cortex) [43]. Provided cTBS has the same effect on LDLPFC GABA levels in patients, this could reduce the severity of positive symptoms in schizophrenia. Another possible protocol is increasing inhibitory tone via low frequency rTMS (e.g., 1 Hz) [48], Whether the inhibitory effect of cTBS and 1 Hz rTMS translates to stronger frontostriatal inhibitory control is an empirical question that could be addressed via DCM in future studies.

### DCM could provide insights on treatment efficacy

Previous works combining rTMS and DCM have shown the feasibility of DCM in describing (non-invasively) the effect of induced brain responses on the Parkinsonian brain [17] and stroke patients [11]. Other connectivity methods could also be used to estimate changes in connectivity parameters of resting state fMRI after stimulation [29]. For example, using functional (i.e., correlational) connectivity analysis Vercammen et al. [49] showed an increase in the correlation between the activity of the LTPJ and the right insula after 6 days of low-frequency (1 Hz) rTMS. However, unlike functional connectivity analysis, DCM would provide realistic biological interpretation of connectivity parameters by explaining the direct (i.e., causal) effects of rTMS on both (within region) inhibitory (i.e., GABAergic) connections and (between region) excitatory (glutamatergic) connections. PEB models of the sort used in this work could also serve as a tool to investigate the variability of the intervention associated with demographic and antipsychotic medication. Finally, at the subject level, DCM could provide timely information to the clinician about the effectiveness of the intervention before symptoms assessment (which is time consuming).

## Limitations

The interpretation of the current results relies on several simplifying assumptions. We focused our analysis on a two-node network which does not represent the actual underlying mechanism of psychosis. For example, the severity of negative symptoms may relate to the LDLPFC’s connectivity with a different seed, not evaluated here. Furthermore, the two-state DCM approach comprises only two neuronal populations per region, which does not represent the actual canonical microcircuit either in the cortex or in the striatum. We focused on a target that is immediately accessible for rTMS and did not study the extended circuitry including the hippocampal excitatory connections that may have a critical “accelerator” effect on the striatum. Individual variability to non-invasive brain stimulation is still poorly understood; our work raises the question of utilizing frontostriatal connectivity parameters to estimate the physiological state of the corticofugal striatal pathways, before choosing the parameters of brain stimulation in schizophrenia. We expect that such an approach will pave way for expanding TMS treatment to hitherto understudied conditions such as delusional disorders and psychotic depression. Finally, although the current model is robust in its explanatory validity, a future work should assess whether the parameters could predict new patients’ total positive symptom score from the connections’ strength of the network (i.e., predictive validity). This could be achieved by using the Leave-One-Out cross-validation^2^ and by studying a larger sample representative of those being evaluated for rTMS.

## Footnotes

1 We thank one of our reviewers for highlighting this limitation.

2 We thank one of our reviewers for suggesting this follow-up work.
